# Effects of Aging and Tai Chi on a Finger-Pointing Task with a Choice Paradigm

**DOI:** 10.1155/2013/653437

**Published:** 2013-02-17

**Authors:** William W. N. Tsang, Jasmine C. Y. Kwok, Christina W. Y. Hui-Chan

**Affiliations:** ^1^Department of Rehabilitation Sciences, The Hong Kong Polytechnic University, Hung Hom, Kowloon, Hong Kong; ^2^Department of Physical Therapy, College of Applied Health Sciences, University of Illinois at Chicago, Chicago, IL, USA

## Abstract

*Background*. This cross-sectional study examined the effect of aging on performing finger-pointing tasks involving choices and whether experienced older Tai Chi practitioners perform better than healthy older controls in such tasks. *Methods*. Thirty students and 30 healthy older controls were compared with 31 Tai Chi practitioners. All the subjects performed a rapid index finger-pointing task. The visual signal appeared randomly under 3 conditions: (1) to touch a black ball as quickly and as accurately as possible, (2) not to touch a white ball, (3) to touch only the white ball when a black and a white ball appeared simultaneously. Reaction time (RT) of anterior deltoid electromyogram, movement time (MT) from electromyogram onset to touching of the target, end-point accuracy from the center of the target, and the number of wrong movements were recorded. *Results*. Young students displayed significantly faster RT and MT, achieving significantly greater end-point accuracy and fewer wrong movements than older controls. Older Tai Chi practitioners had significantly faster MT than older controls. *Conclusion*. Finger-pointing tasks with a choice paradigm became slower and less accurate with age. Positive findings suggest that Tai Chi may slow down the aging effect on eye-hand coordination tasks involving choices that require more cognitive progressing.

## 1. Introduction

Eye-hand coordination requires skillful and integrated use of the eyes, arms, hands, and fingers in goal-directed precision movements [1]. The more complicated the stimulus presented and the more decisions to be made, the slower will be the reaction time of persons of any age [2]. Studies have shown that reaction time slows with advancing age, and the slowing-down becomes more pronounced as the task difficulty increases [2, 3]. Other investigators have suggested that increases in task complexity would lead to increases in the demand on central processing resources. Since older adults have fewer available resources for processing information in the brain, their performance could be affected more than that of younger adults when task complexity increases [3]. Thus, age-dependent-task complexity effect is probably due to age-induced changes in the cognitive processing resources of the central nervous system.

Tai Chi, a mind-body exercise, has a long history and is now practiced by millions of older adults both in the East and the West. Performing its 108 forms amounts to complex motor skill training [4] and requires a great deal of eye-hand coordination and balance control. Previous findings in our laboratory have shown that experienced Tai Chi practitioners display significantly better accuracy than controls similar in age, gender, Mini-Mental Status Examination scores, and physical activity level in finger pointing toward stationary signals appearing contralaterally and centrally to their pointing hand [5]. The practitioners also demonstrated significantly better accuracy when the visual target was moving. Of special interest is that their accuracy was similar to that of much younger controls [5]. 

Previous studies have shown that exercise could improve cognitive functioning in addition to physical performance. This possibility is of particular interest owing to the increased prevalence of cognitive deficits in the aging population [6]. Tai Chi requires its practitioners to incorporate deep and rhythmic breathing as well as mental concentration [4, 7]. Its practice has been demonstrated to improve relaxation, emotional and psychological status [8]. Our own previous investigations have demonstrated that Tai Chi practitioners displayed significantly better attention and memory than healthy control subjects and practitioners of less cognitively demanding aerobic activities [9]. Therefore, the objectives of the present study were to examine (1) the effect of aging on finger-pointing tasks with a choice paradigm that required more cognitive processing and (2) the extent to which experienced Tai Chi practitioners demonstrated better performance than controls similar in age, height, gender, and physical level.

## 2. Methods

### 2.1. Participants

30 young university students (aged 24.2  ±  3.1 years) were compared with 30 healthy older control subjects (aged 72.3 ±  7.2 years) and 31 experienced (mean = 7.1  ±  6.5 years of practice) Tai Chi practitioners (aged 70.3  ±  5.9 years) in this cross-sectional study. Students were recruited from a local university, while older control subjects were recruited from several community elderly centers. The latter had no previous experience in Tai Chi, though some claimed to take regular morning walks or do stretching exercises. To be included in the Tai Chi group, subjects had to practice Tai Chi more than 1.5 hours per week for 3 years or more. All older subjects were subjected to 4 screening tests. They had to (1) score at least 24 in the Mini-Mental Status Examination (MMSE) to exclude cognitive deficits [10, 11]. They also had to (2) attain 20/20 or above in Snellen's visual acuity test [12], with eye glasses if necessary; (3) demonstrate sufficient active range of motion in their upper limbs to perform the finger-pointing tasks; and (4) complete a modified Minnesota Leisure Time Physical Activity Questionnaire [13–16]. 

Subjects with any eye pathology such as glaucoma or cataract which affected the finger pointing test were excluded, as well as those suffering from any pathology affecting their upper limb function such as stroke, Parkinson's disease, or any disabling neurological or musculoskeletal disorder. Other exclusion criteria were peripheral neuropathies of the upper extremities or metastatic cancer. The project was approved by the Ethics Committee of The Hong Kong Polytechnic University, and written informed consent was obtained from all subjects before the study began.

### 2.2. Test Procedures

**Figure 1:**
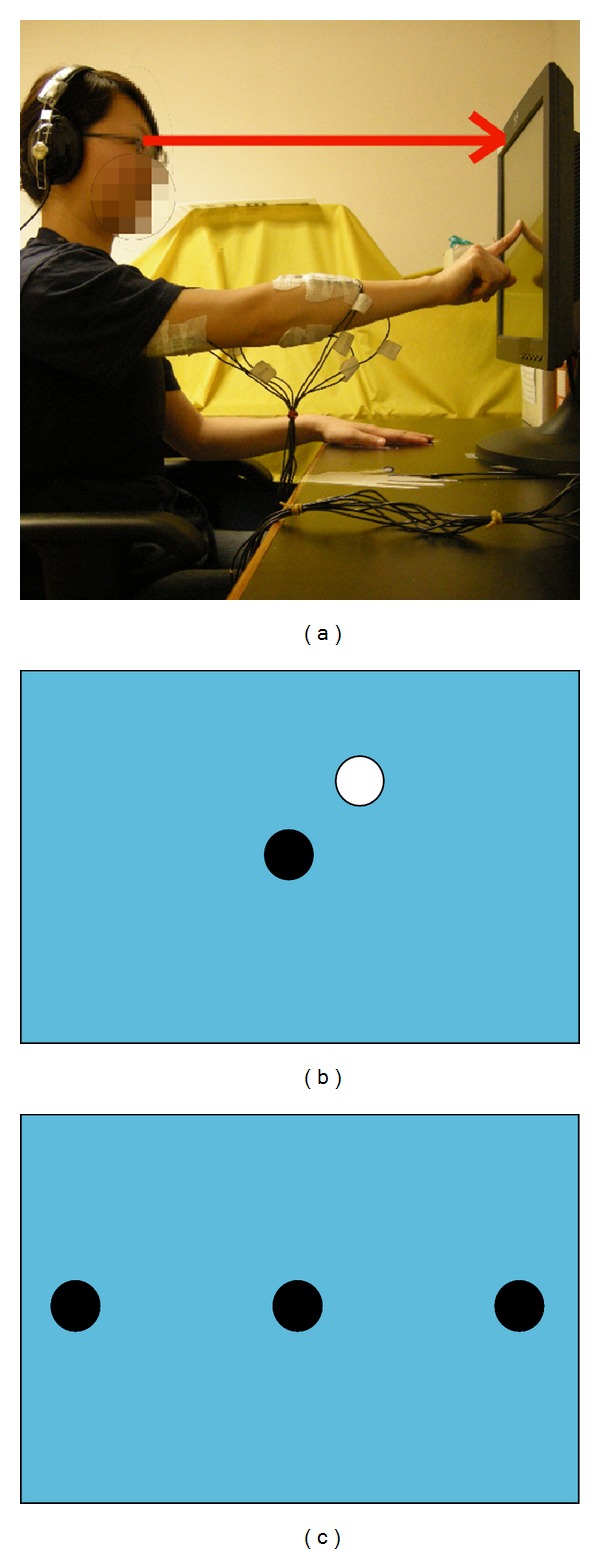
(a) The experimental setup. (b) Condition 3 with the appearance of white and black balls (not drawn to scale). (c) Visual signal locations (not drawn to scale).

Subjects were instructed to point with the index finger of their dominant hand (used for writing or holding chopsticks) as quickly and as accurately as possible, from a fixed starting position on a desk to a visual signal appearing on a vertical display unit (3M Touch Systems; 3M Optical Systems Division, 300 Griffin Brook Park Drive, Methuen, MA 01844, USA). The visual display unit was fixed on and perpendicular to the supporting surface, with its upper edge at each subject's eye level 36 cm from the supporting surface. To start, subjects' index finger rested on the desktop 10 cm from the screen. The visual signal was a ball with a 1.2 cm diameter. Subjects sat still on a height-adjustable chair with the arm rests in front of a computer-controlled LCD touch screen, with their hands resting on a table and their elbows, hips, knees, and ankle joints positioned at about 90°. The chair height was adjusted so that the upper edge of the visual display unit was at the subject's eye level. A mark was positioned at the center of the upper edge of the display unit, and subjects were asked to fixate their eyes on it during the testing (Figure 1(a)). Their upper trunks were strapped to the chair with a Velcro belt to prevent trunk movement. This is because finger pointing can involve either trunk and arm movement or arm movement alone [17]. Since data collection involved recording electromyographic (EMG) responses of arm muscles only, it was necessary to inhibit trunk movement so that subjects pointed to the visual target with only arm movement. 

A visual signal appeared randomly under 3 conditions: (1) a black ball required the subjects to touch it as quickly and as accurately as possible; (2) a white ball required the subjects not to touch it; (3) both black and white balls required subjects to touch only the white ball but not the black ball (Figure 1(b)). The LCD panel was 34 cm wide and 27 cm tall and was divided into 1000 (unmarked) sections from left to right and from top to bottom. The visual signals that required touching appeared at positions 100, 500 (middle-left) 500, 500 (centre), and 900, 500 (middle-right) of the LCD monitor (Figure 1(c)). Conditions 1 and 3 appeared 15 times each and condition 2 for 10 times. Thus, there were a total of 40 runs appearing in a random order for each subject. Each set of coordinates was touched 10 times. Familiarization trials were given for each condition before data recording, to ensure that subjects understood how to perform the task.

### 2.3. Data Recording and Analysis

A pair of surface electrodes was used to record EMG activity in the anterior deltoid muscle (prime mover for arm reaching movement component) [18] of subjects' dominant arm. The electrode pair was attached with electrolyte gel and adhesive tape along the muscle as recommended by Cram and Kasman [19]. EMG signals were recorded using stainless steel surface electrodes (B & L Engineering, 1901 Carnegie Ave, Ste Q, Santa Ana, CA 92705 USA; interelectrode spacing = 1.375 in (3.493 cm)), amplified with a gain of 320 and a total input impedance of more than 100 mega Ohms over a bandwidth of 12 Hz to 3000 Hz. They were sampled at 1,000 Hz and were stored for off-line analysis using an analog/digital converter card (National Instruments, 11500 N Mopac Expwy, Austin, TX 78759-3504, USA). These EMG signals were subsequently processed using the LabView software suite. They were full-wave rectified and smoothed using a second-order Butterworth low pass filter with a cut off frequency of 10 Hz. 

Four measures, namely, reaction time, movement time, end-point accuracy, and number of wrong movements were used to compare among the 3 groups. (1) Reaction time (RT) was the time from the appearance of the ball on the screen to the onset of the anterior deltoid EMG response, defined as the time point when the EMG signal deviated more than 3 standard deviations from the baseline. This point was determined using a tailor-made LabView software program and then visually verified each time. (2) Movement time (MT) was defined as the time from the onset of the EMG response to the time when the ball was touched, called “end point” in this study. This included the time required for muscle torque generation to complete the pointing task. By convention, EMG movement time is defined as the interval from the onset to the end of the EMG signal. Because older adults displayed longer biomechanical delay due to muscle atrophy and related neuromuscular degeneration with age [20], we included the time for generating the muscle torque required to complete the pointing task, for comparison of the MT across the 3 cohorts in this study. (3) Precision in locating the ball on the LCD screen, termed “end-point accuracy”, was defined as the absolute deviation of the subject's finger pointing location from the center of the ball. (4) The number of wrong movements was defined as touching either the white ball in condition 2 or the black ball in condition 3.

### 2.4. Statistical Analysis

To ensure data reliability, an intraclass correlation coefficient (ICC) was used to assess the test-retest reliability of the outcome measures. One-way analysis of variance (ANOVA) was used to compare age, height, and arm length among the 3 groups, and gender was compared using a chi-square test. Since the starting position of the hand and the visual display unit was fixed for all participants, any differences in arm length might constitute a covariate in finger-pointing tasks that involved cognitive processing in a choice paradigm. Therefore, subjects' arm length would be treated as a covariate in the statistical analysis, if a significant difference were found. Arm length was defined as the distance between a subject's acromion and the tip of the middle finger. For comparisons between the 2 older groups, independent *t*-tests were conducted with the MMSE scores, and a chi-square test was used for comparisons of older subjects' physical activity level. Multivariate analysis of variance (MANOVA) was used to compare each of the outcome measures, namely, RT, MT, and end-point accuracy, among the 3 groups and the 3 locations. If statistically significant differences were found in the multivariate tests, univariate tests were conducted for each of the locations. *Post hoc* analysis using Bonferroni's adjustment was conducted if a significant difference was found in the univariate test. One-way ANOVA was used to compare the number of wrong movements among the 3 groups. If a statistically significant difference was found in the one-way ANOVA, *post hoc* analysis using Bonferroni's adjustment was conducted. A significance level (*α*) of 0.05 was chosen for the statistical comparisons.

## 3. Results

### 3.1. Subjects

80 elderly subjects volunteered to participate in this study. Two Tai Chi practitioners were excluded because they had less than 3 years of practicing Tai Chi. Among the control subjects, 3 were excluded because of previous Tai Chi experience; 8 were excluded due to MMSE scores lower than 24; and a further 6 subjects were excluded because they were unable to score 20/20 or more in Snellen's acuity test.

Table 1 shows significant differences in the age, height, and arm length between young control and older (control and Tai Chi) subjects. Arm length, being a confounding variable in finger pointing tasks, was treated as a covariate in the MANOVA statistical analysis. The results in Table 1 also demonstrate that Tai Chi practitioners and older control subjects were similar with respect to age, height, arm length, gender, MMSE scores, and physical activity levels.

### 3.2. Test-Retest Reliability

Among the 61 older adult participants, 6 males (3 Tai Chi subjects) and 14 females (9 Tai Chi subjects) with a mean age = 69.2 ± 7.1 years returned to the laboratory 1 week after the first finger-pointing trials for a second assessment. The ICC values for the RT, MT, and end point accuracy were 0.66 (confidence interval 0.41–0.80), 0.85 (confidence interval 0.74–0.91), and 0.68 (confidence interval 0.41–0.82), respectively, which indicated moderate-to-satisfactory reliability. The ICC value for the number of wrong movements was 0.46 (confidence interval −0.51–0.80). This was considered as fair reliability and should be treated with caution.

### 3.3. Finger-Pointing Tasks with a Cognitive Component

Young university students showed significantly faster RT and MT times, with significantly greater end-point accuracy and fewer wrong movements than older control subjects in finger pointing to all 3 target locations (*P* < 0.05 for all, Table 2). Tai Chi practitioners achieved a significantly faster MT and made fewer wrong movements in finger pointing to all 3 locations than older control subjects. They also demonstrated significantly better end-point accuracy than older controls, when the visual targets appeared contralateral to their pointing hand. Of special interest is that RT in finger pointing to the center and ipsilateral target locations, end-point accuracy, and number of wrong movements were even similar to those of young subjects (Table 2). 

## 4. Discussion

### 4.1. Effects of Aging

Because many situations in daily life require people to set priorities and choose responses to different signals, we have chosen to study a finger-pointing task involving a choice paradigm in this study. Our results demonstrate that eye-hand coordination in a finger-pointing task with a choice paradigm declines with age. These findings agree with those of our previous investigations on the effects of aging on finger-pointing tasks with stationary and moving targets [5] and with other investigations on eye-hand coordination [3, 21]. Moreover, with the addition of a choice paradigm, we found that declines in eye-hand performance were comparatively greater in older than younger control subjects, as it will emerge below.

Compared with a simple and fast finger-pointing task using a stationary visual signal with the same experimental set-up in our previous study [5], both young university students and older control adults had slower RT and MT when a choice paradigm was added to the finger-pointing task in our present study. Using the central visual target location (500, 500) as an example, the RT and MT of the young students in the simple task were 289.6 ms and 583.2 ms [5], while the same measures using a choice paradigm were 431.6 ms and 617.9 ms (Table 2), representing an increase of 49% and 6%, respectively. However, the within-group increases in RT and MT for the older adults were more than those for the young subjects, being 60% (322.2 ms [5] versus 515.1 ms) for RT and 29% (768.7 ms [5] versus 990.1 ms) for MT. The young subjects' end-point accuracy in touching the center of the visual target was similar in simple and choice conditions, being 7.28 mm and 7.33, respectively, with an increase in error of only 0.7%. However, the older adults performed more poorly in the choice paradigm, showing an error of 16.8 mm (Table 2) compared with 12.8 mm in the simpler task [5], which represented an increase of 31%. The cognitive demand introduced in a choice paradigm appeared to have affected performance of finger-pointing tasks to a greater extent in the older than the young subjects. Fitts' law describes characteristics of arm movements using the equation MT = *a* + *b* log⁡_2_ ⁡2*D*/*W*, where MT represents the movement time, *D* the distance moved, and *W* the width of the target, with *a* and *b* as constants [22]. Such an equation has not taken into consideration any cognitive component of the task, nor the effect of aging as shown in our previous [5] and present cross-sectional studies between young and older subjects.

### 4.2. Effects of Tai Chi Practice

#### 4.2.1. Movement Time

Tai Chi practitioners achieved significantly faster MT in finger pointing to all 3 target locations than those of older controls (Table 2). This differs from our previous findings that both older groups demonstrated similar MT when they performed simple and fast finger pointing to stationary and moving visual targets in a no-choice situation [5]. The latter finding that both groups could reach stationary visual targets with similar MT [5] would have precluded possible between-group differences in musculoskeletal and neuromuscular factors, including range of joint movement and muscle strength in that task. However, in addition to these factors, eye-hand coordination also involves perceptual, cognitive, and motor staging factors [3, 23, 24]. Shumway-Cook and Woollacott [20] detailed the key cognitive skills in eye-hand coordination tasks as “problem solving, selective attention, planning, memory, and intention, among others.” While one can reach for objects with neuromuscular capability alone, “one's ability to acquire a range of solutions for difficult tasks and correctly identify the usefulness of objects is affected by cognition.” 

The mean MT that Tai Chi practitioners needed to touch the visual target in the central location (500, 500) was 762.3 ms in the stationary protocol [5]. It increased only slightly to 845.2 ms in the choice paradigm, an increase of just 11% (Table 2). This is much less than the increase of 29% reported previously for the older control subjects. The difference in the findings between the 2 studies on simple and choice paradigms may be explained in terms of greater cognitive skills being required to perform eye-hand coordination in a choice paradigm [3, 24]. 

#### 4.2.2. End-Point Accuracy

Tai Chi practitioners demonstrated better end-point accuracy than older control subjects in pointing to a contralateral visual signal (average errors of 12.7 mm versus 24.3 mm for middle left target location, resp.), even though they were moving significantly faster (MT = 886.5 ms versus 1032.6 ms; Table 2). Previous study had shown that 8 weeks of Tai Chi training enabled older adults (average age = 78.8 years) to achieve smoother movements, showing fewer jerky movements than those involved in a walking or jogging program, when they were instructed to move a stylus with their dominant hand to reach a final target by crossing another intermediate target with a curved line [25]. In a previous cross-sectional study, we also found that experienced Tai Chi practitioners were significantly more accurate in pointing to stationary and moving visual signals than older controls similar in age, gender, and physical activity level, and their performance was even similar to that of young university students [5]. Tai Chi requires the practitioners to follow their hand movements closely with their eyes. Such specific eye-following-hand training may help to enhance eye-hand coordination in the practitioners [5, 26]. Also, Tai Chi practice puts great emphasis on both exact joint positioning and direction of movement. In fact, previous findings from our laboratory have shown that experienced Tai Chi practitioners had better joint proprioception [13]. It should be noted that joint proprioception has been shown to be an important source for accurate reaching movements [27, 28]. Taken together, these factors could have led to improved end-point accuracy of the finger, despite the use of a choice paradigm requiring more cognitive processing.

#### 4.2.3. Wrong Movements

Table 2 showed that Tai Chi practitioners made significantly fewer wrong movements than older controls. Such a finding may be due to their better attention in the signal encoding process and their better registration of the visual signal in the memory retrieval process than control subjects, as shown by our previous study [9]. A possible explanation is that Tai Chi practice requires the practitioners to pay attention to and to recall a long sequence of movements, usually from 24 to 108 forms.

Since this study used a cross-sectional design, a causal relation between Tai Chi practice and better finger-pointing performance could not be established. A longitudinal study would be required to establish the causal relationship between the two. Because only healthy older adults were examined, the results cannot be extrapolated to younger or frail older individuals or to those who have a history of visual or cognitive problems. 

## 5. Conclusions

Limitations aside, the findings from this cross-sectional study suggest that experienced Tai Chi practitioners may function better than older controls in daily activities that require eye-hand coordination to reach an object of choice, such as reaching for a spoon located amongst an assortment of cutleries or for the needed key in a bunch of keys. Worthy of note is that their RT in finger pointing to the central and ipsilateral locations of a visual signal in a choice paradigm, end-point accuracy, and number of wrong movements were even similar to those of the young control subjects.

## Figures and Tables

**Table 1:** Comparisons of age, height, and arm length among young control, older control, and older Tai Chi subjects and of Mini-Mental Status Examination and physical activity levels between the two older groups.

	Young control subjects	Older control subjects	Tai Chi subjects	*P*
	(*n* = 30)	(*n* = 30)	(*n* = 31)	
Age (y)	24.2 ± 3.1	72.3 ± 7.2^†^	70.3 ± 5.9^†^	<0.001*
Height (m)	1.67 ± 0.08	1.58 ± 0.08^†^	1.59 ± 0.07^†^	<0.001*
Arm length (cm)	70.5 ± 4.3	65.3 ± 5.8^†^	67.2 ± 4.3^†^	<0.001*
Gender (M/F)	15/15	15/15	15/16	0.989
MMSE score	—	26.7 ± 2.0	26.6 ± 1.9	0.966
Physical activity level				0.364
Light < 4 METs	—	*n* = 15	*n* = 10	
Moderate 4–5.5 METs	—	*n* = 11	*n* = 16	
Heavy > 5.5 METs	—	*n* = 4	*n* = 5	

Note. Values are mean ± SD for this and all subsequent tables.

Abbreviations: F: female; M: male. MMSE: Mini Mental Status Examination; MET: metabolic equivalent.

—: denotes “There was no need to record the data.”

*denotes significant difference at *P* < 0.01 level using one-way ANOVA.

^†^denotes significant difference from the young controls at *P* < 0.05 level by means of *post hoc* analysis using Bonferroni's adjustment.

**Table 2:** Comparison of anterior deltoid reaction time, movement time, end-point accuracy, and number of errors in finger pointing with a choice paradigm.

	Young control subjects	Older control subjects	Tai Chi subjects	*P*
	(*n* = 30)	(*n* = 30)	(*n* = 31)	
EMG reaction time (ms)				
Middle left	430.8 ± 90.9	569.4 ± 156.5^†^	522.2 ± 126.3^†^	<0.001**
Center	431.6 ± 92.0	515.1 ± 109.8^†^	488.6 ± 99.0	0.006**
Middle right	447.1 ± 92.5	568.8 ± 159.1^†^	518.8 ± 97.0	0.001**
EMG movement time (ms)				
Middle left	660.0 ± 116.4	1032.6 ± 284.7^††^	886.5 ± 183.5^†^	<0.001**
Center	617.9 ± 115.9	990.1 ± 250.2^††^	845.2 ± 167.6^†^	<0.001**
Middle right	585.6 ± 103.0	942.2 ± 206.9^††^	801.9 ± 159.1^†^	<0.001**
End-point accuracy (mm)				
Middle left	9.4 ± 2.5	24.3 ± 25.8^††^	12.7 ± 9.8	0.002**
Center	7.3 ± 2.4	16.8 ± 22.8^†^	11.4 ± 8.3	0.040**
Middle right	9.9 ± 3.9	16.7 ± 13.8^†^	11.6 ± 8.4	0.022**
Wrong movement (number)	0.2 ± 0.5	3.3 ± 6.0^††^	1.0 ± 1.5	0.003*

*denotes significant difference at *P* < 0.01 using one-way ANOVA.

**denotes significant difference at *P* < 0.05 using univariate tests, after multivariate ANOVA showing *P* < 0.05.

^†^denotes significant difference from young controls at *P* < 0.05 by means of *post hoc* analysis using Bonferroni's adjustment.

^††^denotes significant difference from young controls and Tai Chi practitioners at *P* < 0.05 by means of *post hoc* analysis using Bonferroni's adjustment.
